# First trend analysis of antifungals consumption in Lebanon using the World Health Organization collaborating center for drug statistics methodology

**DOI:** 10.1186/s12879-022-07883-5

**Published:** 2022-11-24

**Authors:** Deema Rahme, Mayssam Ayoub, Khalil Shaito, Nadine Saleh, Sara Assaf, Nathalie Lahoud

**Affiliations:** 1 Pharmacy Practice Department, Faculty of Pharmacy, Beirut Arab University, Riad El Solh 11072809, P.O. Box 11-5020, Beirut, Lebanon; 2 Pharmacy Department, Rizk Hospital, Lebanese American University Medical Center, Beirut, Lebanon; 3 Faculty of Pharmacy, Lebanese University, Hadat, Lebanon; 4 Pharmacy Department, Saint George Hospital, Hadat, Lebanon; 5 Faculty of Public Health, Lebanese University, Fanar, Lebanon; 6 INSPECT-LB: Institut national de santé publique, épidémiologie clinique et toxicologie-Liban, Beirut, Lebanon

**Keywords:** Antifungal agents, Fluconazole, Drug resistance, Antimicrobial stewardship, Community pharmacy

## Abstract

**Background:**

Antimicrobial resistance has reached an alarming rate globally, especially in middle-income countries such as Lebanon. The development of antifungal resistance is associated with the increased population’s injudicious consumption. This study aims to measure antifungals consumption in Lebanon as a trend analysis of national data from 2004 to 2018.

**Methods:**

This is a trend analysis of the consumption of antifungal agents in the Lebanese community. Data were obtained from the Intercontinental Marketing Statistics Database between 2004 and 2018. It measures the total consumptions per year, per drug, and the percentage of its correspondents for three routes of administration (oral, parenteral, and topical). Results were reported by Defined Daily Dose (DDD) per 1000 inhabitants per day and the total number of DDDs.

**Results:**

Community consumption of antifungals in Lebanon has increased by approximately 18.64% between 2004 and 2018, as measured by the number of DDDs per 1000 inhabitants per day; and amplified by approximately 87.76% as measured by the number of DDDs. The highest consumption level was noted in 2017, with 1.52 DDDs/1000 inhabitants/day and 3,386,930 DDDs. Fluconazole was the most consumed antifungal while micafungin was the least with 6,723,869.2 (20.99%) and 48.5 (0.0002%) DDDs respectively. Topical antifungals ranked the first type consumed followed by oral and parenteral antifungals representing 51.72%, 48.24%, and 0.033% of the total consumption respectively.

**Conclusion:**

The findings from this study indicate a marked increase in antifungal consumption in the Lebanese community. This accelerates the need of implementing disease management guidelines and national antifungal stewardship. Moreover, these findings may be used in further benchmark utilization and antimicrobial resistance studies in Lebanon.

Impact on practice

- This study raises an urgent demand for the development and implementation of a national antimicrobial stewardship program.
- Healthcare providers including physicians and pharmacists have a pivotal role in promoting the prudent use of antifungals.
- Pharmacists should reduce non-prescription antifungal dispensing and refer to physicians accordingly.
- Pharmacists are responsible for raising community awareness about the proper use and indications of antifungals.

## Introduction

Infections are a major concern for multidisciplinary teams across the world [1]. In the last three decades, there was a remarkable ascent in the incidence of fungal infections varying according to patient factors, environmental causes, and antifungal exposure [1, 2].

Invasive fungal infections (IFIs) are significant in hospitals and communities due to their emergent prevalence, high morbidity and mortality, and healthcare-related costs [3]. Mostly, immunocompromised conditions put patients at high risk of severe or even lethal fungal infections [4].

A large US healthcare network revealed that the mean incidence of IFIs was 27.2 cases per 100,000 patients per year whereas *Candida* accounted for 55.2% of the cases [5]. However, 42-day mortality was highest in *Aspergillus spp*. (27.5%), followed by *Candida* (20.5%), and lowest for dimorphic fungi (7.5%) [5]. In the United Kingdom, more than 5,000 cases of invasive *Candida* infections take place each year [6].

Moreover, Global Antifungal Drug Market expected that the $14.27 billion cost in 2017 would grow by more than 3.85% between 2018 and 2025 [7].

Despite the availability of many antifungal agents, their judicious use is not achieved in most cases [3, 8]. The increase in the consumption of antifungal agents creates a favorable environment leading to the increased selection of resistant fungal strains.

According to a systematic review, resistance rates reported were between 11.9% and 14% for *Candida glabrata* and over 2.3% for *Aspergillus fumigatus* [9]. Besides, another study showed that 11.9% of *Candida. glabrata* and 11.6% of *Candida tropicalis* were resistant to fluconazole [10]. Furthermore, according to the Centers for Disease Control and Prevention (CDC), *Candida* isolates were constantly resistant to fluconazole over the past 20 years and their resistance to echinocandins is rising as well [11]. Azole resistance among Candida and Aspergillus species is one of the greatest challenges to treatment success, followed by echinocandin and multidrug resistance among some Candida species, especially *Candida glabrata *[12]. The extent of the emergence of resistance is associated with antifungal consumption volumes [13]. Therefore it is crucial to assess antifungals utilization to understand the epidemiology of resistance and to implement effective antifungal stewardship programs to control antifungal drug resistance,

In the Middle East, studies tracking the trends of antifungal consumption and evaluating the correlation between their usage and the emergence of resistance remain scarce [2]. A recent review on the epidemiology of *Candida* species in the Middle East and North Africa (MENA) region revealed, as witnessed worldwide, an evident shift of *Candida albicans* towards non-*albicans Candida*. This finding is alarming for the emergence of multi-drug resistant *Candida* species in the MENA region [14]. In Lebanon, few studies assessed antifungal consumption. A study at Al-Makassed Hospital revealed that the total consumption of antifungals was 180.69 ± 125.5 Days Of Therapy/1000 Patient Days with the highest consumption in the hematology/oncology department and lowest in the obstetrics/gynecology department between 2008 and 2015 [15]. Azoles were the most common first-line antifungals, whereas echinocandins and amphotericin B were mostly prescribed in the hematology/oncology department [15].

Another retrospective study at the American University of Beirut Medical Center revealed 1300 and 1500 *Candida* isolates per year [16]. Fluconazole was active against *Candida albicans* whereas voriconazole and amphotericin B had a consistent activity against the most recovered *Candida* isolates [16].

This study’s objective was to assess the national consumption trend of antifungals in Lebanon. Measuring the rate of consumption of antifungals helps identify areas with a high threat of the spread of AMR. Moreover, the results from this study help to bring awareness of the factors related to the increased antifungals consumption, which serves as a baseline in the AMS program. In addition, it will be used in future studies on the surveillance and monitoring of antifungal resistance in Lebanon by comparing the consumption trends with the reported levels of antifungal resistance trends.

## Methods

### Study design and setting

This is a trend analysis study estimating the consumption of antifungal agents as per DDD on community data, from 2004 to 2018.

### Data source

The national consumption of antifungal agents was obtained from the Intercontinental Marketing Statistics (IMS) Health database in Lebanon. It provides consumption data based on the volume of sold antifungals in community pharmacies, through national surveys conducted by the pharmaceutical distributing agents. The consumption is presented in units of boxes for each product and its corresponding dosing and dosage form. The data obtained were from 2004 to 2018 yet 2016 figures were missing since the data was a combination of both hospital and community this year. The number of inhabitants per year was obtained from the World Bank data for calculating the consumption by DDD/1000 inhabitants/Day [19].

### Inclusion and exclusion criteria

Sales data including the number of boxes sold for all antifungal drugs in the Lebanese community pharmacies during the study period were included to assess national antifungal consumption in the Lebanese community. However, data concerning antifungal drug consumption and sales in hospitals were excluded from the study.

### Data analysis

The IMS database was obtained for different years, containing 120 available antifungals in the Lebanese market. It lists the medication name provided with its dosage form, dose strength, number of units in each box, and the total number of boxes consumed in the community for each drug in a specific year from 2004 to 2018. Moreover, the Anatomical Therapeutic Classification (ATC) code following the World Health Organization (WHO) classification of each active ingredient/route of administration was added [17]. For each drug and route of administration, the following calculation was performed every year. First, we multiplied the dose strength of one box by the number of units per box to obtain the total dosing of the whole box. Then, the result is multiplied by the number of boxes consumed per year. Finally, the latter calculated field was divided by the specific ATC/DDD value to acquire the number of DDDs consumed annually. To obtain DDD/1000 inhabitants/day, the annual number of DDDs was divided by the population size that year and 365, then multiplied by 1000.

The data was presented in tables to depict the total number of DDDs and DDDs per 1000 inhabitants per day, for each active ingredient from 2004 to 2018 including “oral”, “parenteral”, and “topical” routes. Data was also sorted by classes of antifungals. All figures were generated and portrayed using Microsoft Excel 2016.

## Results

The Lebanese population size was 6,859,408 and consists of approximately 50% males (3,449,884). The majority of the population is adults, with a median age of 28 years old, whereas elderlies above 65 years old and children below 18 years represent 7% (480,321) and 25% (1,742,580) of the total population respectively. The refugee population is approximately 21% [18].

**Fig. 1:**
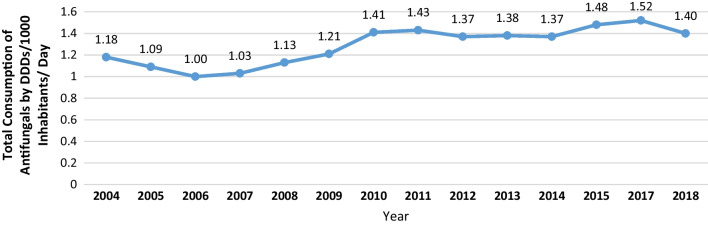
Total consumption of antifungals by DDDs/1000 inhabitants/day between 2004 and 2018

Community consumption of antifungals in Lebanon has generally increased by approximately 18.64% between 2004 and 2018, as measured by the number of DDDs per 1000 inhabitants per day (Fig. 1). The overall trend showed an initial drop of 15.25% between 2004 and 2006 and then continuously increased till 2011. During the 2011 and 2014 periods, there was a somehow stable consumption. Afterward, the highest recorded value was in 2017 with 1.52 DDDs/1000 inhabitants/day. The latter was higher than in 2004 and 2014 consumption by 28.8% and 10.94%, respectively. Finally, there was a slight decrease again between 2017 and 2018 by 7.89%.

The total consumption of each antifungal drug in different classes during the 14 years was shown in Table 1. Fluconazole was the first rank and micafungin was the last with 6,723,869.2 (20.99%) and 48.5 (0.0002%) DDDs, respectively.

**Table 1:** The consumption of antifungal drugs in defined daily doses (DDD) and their percentage consumption from the corresponding antifungal class and the total antifungals consumption between 2004 and 2018

Antifungal class	Allylamines		Azoles											
Antifungal drug	Amorolfine	Naftifine	Clotrimazole	Econazole	Fenticonazole	Fluconazole	Isoconazole	Itraconazole	Ketoconazole	Omoconazole	Posaconazole	Sertaconazole	Miconazole	Voriconazole
Total consumption in DDD	32,610	310,539	5,261,955	520,317	1,841,725	6,723,869	70,913	4,005,650	2,279,202	29,097	7,644	209,932	2,570,771	48,587
Percentage of antifungal class consumption	9.50%	90.50%	22.33%	2.21%	7.81%	28.53%	0.30%	16.99%	9.67%	0.12%	0.03%	0.89%	10.91%	0.21%
Percentage of total antifungals consumption	0.1%	0.97%	16.42%	1.62%	5.75%	20.99%	0.22%	12.5%	7.11%	0.09%	0.02%	0.66%	8.02%	0.15%

Antifungal class	Polyenes		Echinocandins			Others	
Antifungal drug	Amphotericin B	Nystatin	Anidulafungin	Caspofungin	Micafungin	Ciclopirox	Griseofulvin
Total Consumption in DDD	4,171	3,293,436	3,708	241	49	2,932,968	1,889,080
Percentage of Antifungal Class Consumption	0.13%	99.87%	92.75%	6.04%	1.21%	60.82%	39.18%
Percentage of Total Antifungals Consumption	0.01%	10.28%	0.01%	0.0008%	0.0002%	9.16%	5.90%

Total antifungal drugs consumption between 2004 and 2018 = 32,036,467 DDD

**Fig. 2:**
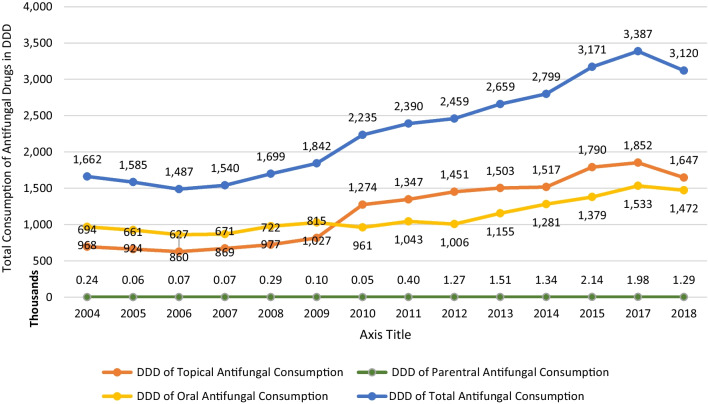
Total consumption of antifungal drugs in defined daily dose (DDD) for oral, topical, and parenteral dosage forms between 2004 and 2018

The trend of oral antifungals consumption follows in most ways that of the total consumption between 2004 and 2018 (Fig. 2). Yet, a steeper increase of 52.34%, compared with the total consumption, was recorded between 2012 and 2017. On the other hand, the consumption of antifungals parentally did not harmonize with that of the total consumption; where it has major variances observed (Fig. 2). The highest recorded value was in 2015 with 2,139 DDDs. No continuous increase was shown throughout the years. Yet, a fluctuation between increase and decrease took place between 2004 and 2018 with a sharp increase between 2011 and 2012 of 215.67% ( Fig. 2). Lastly, the consumption of topical antifungals depicts, mostly, the same trend as that of the total antifungals consumption; with 2017 recording the highest value of 1,852,003 DDDs (Fig. 2 ).

**Fig. 3:**
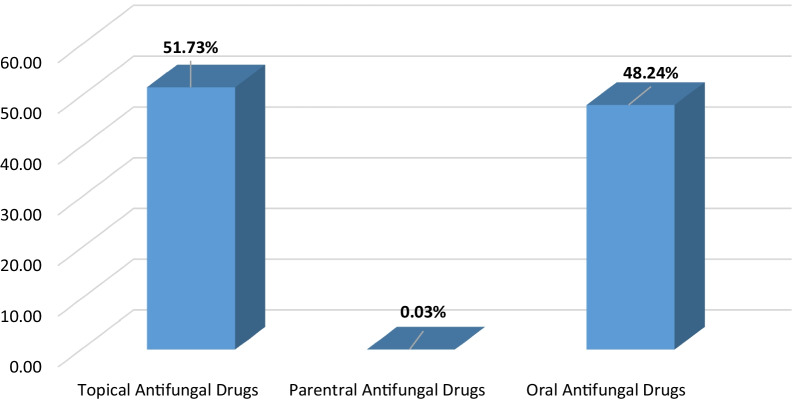
The proportion of topical, parenteral, and oral dosage forms of antifungal drugs utilized between 2004 and 2018

It was also noted that topical antifungals ranked the first consumed dosage form representing 51.73% of total antifungal consumption between 2004 and 2018 followed by oral and parenteral antifungals which represented 48.24% and 0.033% of the total consumption respectively as illustrated in Fig. 3.

## Discussion

This study is the first in Lebanon to analyze the trend of antifungal consumption based on national data recorded between 2004 and 2018. These data indicate an overall increase in antifungal consumption by 18.64%, from 1.18 to 1.4 DDDs per 1000 inhabitants per day. However, a significant increase was measured by the total number of DDDs number which ascended from 1661 thousand to 3120 thousand DDDs. This uptrend is mainly contributed by the overall dominance of azole antifungals (73.57%) generally, and fluconazole (20.99%) specifically. The latter use was amplified by 287.64% between 2004 and 2018.

Results showed an initial slight decline between 2004 and 2006 in antifungal consumption using both measures (DDDs and DDDs per 1000 inhabitants per day). However, this decline was more obvious in the second measure since it was related to the number of current inhabitants during this period. Lebanon was facing political problems during these years. Many citizens left the country so the number of inhabitants decreased as well as the total consumption of drugs including antifungals.

The rates of consumption of antifungals in Lebanon are somehow similar to those in Europe. According to the ESAC reports published between 2010 and 2017, the trend of consumption from 2010 to 2012 showed the same maximum consumption of 3.3 DDDs per 1000 inhabitants per day [19]. This rate increased between 2013 and 2014 to a wider range of 0.36 to 3.8 DDDs per 1000 inhabitants per day [19]. Our study showed a stable consumption, with the lowest value among these years in 2014 (1.37 DDDs/ 1000 inhabitants/ day). Nonetheless, the major discrepancy was the low value of antifungal consumption in Europe during 2017 (0.9 DDDs /1000 inhabitants/ day) in contrast with the maximum value in Lebanon in the same year (1.52 DDDs/1000 inhabitants/ day) [19]. The difference between Lebanon and the European countries in terms of antifungal consumption could be correlated to the social or cultural differences, and variations in the healthcare system, pharmaceutical market, and regulatory systems and resources [20]. Nevertheless, the study findings were similar to the utilization trends of antifungals in Tanzania from 2010 to 2017 which showed a significant increase in the consumption of antiviral antifungal agents [21].

Our study highlighted a rise in antifungal consumption in Lebanon between 2004 and 2018 at 87.76%. Numerous factors contribute to this upward trend. First, similar to antibiotics, a wide range of antifungals is commercially available to the population; in both topical and oral forms (creams, tablets, syrups, etc.) [22]. The market size of antifungal drugs is growing with the increase in over-the-counter (OTC) drugs, especially for dermal use [22]. Second, people usually are self-medicated and can be provided with low-price and prescription-free antifungals from Primary Health Care Centers (PHCCs) [23–25]. Third, unspecialized physicians could be prescribing antifungals without proper implementation of guidelines for disease management [24–26]. Fourth, pharmacists participate in dispensing medications such as antibiotics without physician prescriptions, and could probably as well dispense antifungals [23–25]. Finally, there is no strict and clear implementation of an authorized protocol for antimicrobial sales by the national regularities [23–25]. Furthermore, the increase in antifungal consumption can be attributed to the increase in cancer cases. Cancer patients are prescribed antifungal therapy as prophylaxis or as treatment and hence, increased cancer incidences according to CDC statistics will magnify antifungal consumption [27]. In addition, an important reason behind the overall increased use of antifungals could be the increasing number of invasive fungal infections such as chronic pulmonary aspergillosis, cryptococcal meningitis in HIV/AIDs, invasive candidiasis, and *Pneumocystis jirovecii pneumonia *[28].

Increased incidences of infections were also powered by the increase in global travel that is accompanied by the spread of fungi wherever people go; as well as the climate change dilemma that allowed the variation in the geographical features and rooting of the fungi [29]. As a fact, global warming would significantly affect the distribution of species that are tolerant to heat by favoring conditions needed by some fungi to spread closely into the human population [30].

Among the interesting findings was the 7.87% decrease in antifungal consumption between 2017 and 2018 which is worth elaborating on. During this period and until nowadays, Syrian refugees are returning home as reported by the Ministry of Social Affairs, United Nations Development Program (UNDP), and the United Nations High Commissioner for Refugees (UNHCR) in the Regional Refugee and Resilience Response Plan (3RP), which could implicate a possible decrease in antifungals’ use [31].

The study results were depicted differently between the three routes of administration. Topical drugs were ranked first having 7.22% and 153.492% higher than the consumption of oral and parenteral drugs respectively. This might be related to the fact that topical antifungals are commercially available in a huge number of brands and forms in community pharmacies, such as creams, sprays, shampoos, and nail lacquers, which could be mostly sold as OTC products. Additionally, topical antifungals are less expensive than other dosage forms. Also, parenteral drugs usually require hospitalization so they are not readily available in the community.

Azoles dominated the overall consumption trend. They are mostly used in clinical practice and are readily available in numerous forms and generics/brands and they are the most studied in terms of mode of action, pharmacological characteristics, and resistance profile [32]. The most consumed antifungal agent was fluconazole. Its use was similar to the trend between Lebanon and other nations. This trend is in agreement with other European countries as reported by the European Centre for Disease Prevention and Control (ECDC). The latter also states that Belgium records the highest fluconazole use within Europe (after Greece in 2016) [33]. Moreover, a study conducted in Catalonia on the consumption of systemic antifungals between 2008 and 2013 showed that fluconazole represented 70.3% of antifungals [34]. Similarly, fluconazole had the highest proportion of utilization for antifungals in Tanzania [21]. All of these findings of the high consumption/prescription of fluconazole contributes to the global spread of microbial resistance to it. The point-prevalence study of antimicrobial use in hospitalized neonates and children showed that 20.5% of 146 *Candida. glabrata* cultures were resistant to fluconazole [8]. In addition, 1846 isolates from 31 countries showed that 11.9% of *Candida glabrata* and 11.6% of *Candida tropicalis* were resistant to fluconazole and reported an increase in fluconazole resistance from 9 to 14% between 2001 and 2007 [10]. The Middle East study showed that 38% of *Candida* isolates were fluconazole-resistant and that 42% of the patients were converted to the second line therapy agents due to decreased responses [2].

Finally, it is worth mentioning the reports of recent studies that severe clinical COVID-19 has increased the risk of invasive fungal infections. COVID-19-associated pulmonary aspergillosis emerged as an important fungal complication in patients with acute respiratory failure. Accordingly, antifungal consumption has increased especially echinocandins, voriconazole, and isavuconazole which may be a contributing factor to antifungal resistance pattern in the upcoming years [35–37].

### Strengths and limitations

The chief strength of this study is being the first to address antifungal consumption on a national level, and can also be considered the first in the Middle East region. Additionally, the data on antifungal use in the community sector was readily available from the IMS database as a source of surveillance information. Moreover, the consumption figures were recorded over a long period. Furthermore, using the ATC/DDD system adds value to the results obtained on antifungal consumption as it serves as the benchmark of data used for comparison with other countries [11]. However, the study has some limitations. First, missing 2016 data contributes to the major drawback of this study. This is because IMS 2016 data is a mix of hospital and community figures and we couldn’t extract community ones only. Second, the comparison of usage with other countries is dictated by prescribing guidelines that vary from one country or region to another; knowing that Lebanon lacks national ones for such practice. Third, the IMS database is a record of the sales of antifungal agents to community pharmacies without taking into consideration the previously mentioned sources such as PHCCs and free mobile clinics. However, the IMS database is the only available resource to conduct drug utilization studies in Lebanon. Finally, there are no abundant data in the literature available to use the number of DDDs as a standard unit of measurement/comparison with other national and international studies. The majority of the studies report antimicrobial consumption by one of the following: DDDs per number of inhabitants, DDDs per patient days, etc. Using the latter units of measurement is significant for comparison purposes among countries and for effective longitudinal analysis of consumption trends [38]. Using these indicators can be questioned when looking for comparisons or analyses of trends that have an increase in both, consumption rates and population size [38]. For that, reporting most of the data by the number of DDDs is an attenuated limitation by the need for our trend analysis to reflect the changing overall trend with a population number change. The exaggeration depicted by this measure was strengthened as well by the fact that a stable profile was revealed by the DDDs per 1000 inhabitants per day when an actual increase was taking place. Moreover, WHO states that it is impossible to define pediatric DDDs because dose recommendations for use in children vary according to age and body weight.

## Conclusion

In conclusion, the study depicted an overall increase in antifungal consumption, over 14 years, in community pharmacies in Lebanon. This raises the pivotal need for the development and implementation of national treatment guidelines and an antifungal stewardship program to limit the emergence of AMR. Finally, pharmacists’ key role in patient counseling can rationally guide the use of antifungal drugs and improve patient safety and outcomes.

## Data Availability

All data generated and analyzed during this study are included in this published article. Any further data can be provided by the corresponding author upon request.
